# COVID-19 and the National Lockdown: How Food Choice and Dietary Habits Changed for Families in the United Kingdom

**DOI:** 10.3389/fnut.2022.847547

**Published:** 2022-05-24

**Authors:** L. Scott, H. Ensaff

**Affiliations:** Nutritional Sciences and Epidemiology, School of Food Science and Nutrition, University of Leeds, Leeds, United Kingdom

**Keywords:** COVID-19, food choice, family food, food environment, food literacy, dietary habits, children, food practices

## Abstract

COVID-19 changed the way families in the UK live, with as yet uncertain impacts to food choice and dietary habits. This study sought to explore food-related experiences and changes to behavior of families with children, during the pandemic. Semi-structured one-to-one interviews with parents (*n* = 20) and, separately, their children (*n* = 22; aged 8–10 years) were conducted. An inductive thematic approach was adopted for the data analysis, and four main themes emerged: commensality; elevated place of food in the home; snacking; and food shopping. Study findings highlighted several changes: some related to increased snacking and more takeaway food; others were more favorable, including spending more time together, increased home cooking, more efficient shopping practices and reduced food waste. Overall, an elevation of the place of food within the home was apparent, alongside enhanced food literacy, and some evidence of the relocalisation of food. This study contributes to the international literature on the impact of COVID-19 and national lockdowns on family lifestyle behaviors, specifically food choice and dietary habits; further research into the longer-term effects of COVID-19 on family food practices is required.

## Introduction

The World Health Organization characterized COVID-19 as a pandemic on 12 March 2020 (1). Following this, many countries implemented measures (such as enforced restrictions on movements and activities) to control the virus's transmission. The UK government announced the first national lockdown period on 23 March 2020; the population was asked to stay at home-with the exception of essential travel, medical or care needs, and daily exercise (2). People were asked, where possible, to work from home and follow social distancing guidelines (2). In the same announcement, people were asked to do food shopping as infrequently as possible, and to use food delivery services where possible (2). All schools were mandated to close (with the exception for the most vulnerable children and children of keyworkers) (3) and schools moved to remote learning. As a result, many children spent their days alongside their parents, and many families became confined to home for much of the time.

The UK government announced that a phased reopening of schools could begin in June (4). However, the cap on class sizes meant that a return to schooling was not possible for all (5), and many children did not return until the new academic year in September 2020 (6). Following a relaxation of restrictions over the summer, COVID-19 case numbers began to rise and the UK was in a second wave, when a second 4-week national lockdown was instigated in early November (schools remained open during this second lockdown). Case numbers continued to rise over the Christmas period however, and the UK government implemented a third national lockdown on 4 January 2021 (7), which again included the closure of schools.

The lockdown measures are likely to have had considerable impact on the nation's day-to-day lives. Similar enforced restrictions in other countries have resulted in changes including those associated with unhealthy lifestyle behaviors in families. A cross-sectional survey of 254 families in Canada reported that families with young children experienced increased snacking, decreased physical activity and increased screen time in both parents and children (8). Interestingly, favorable changes were also observed, such as eating less fast food, spending more time cooking and preparing more meals from scratch, as well as eating together with children more often and involving children in meal preparation (8). Likewise, another survey of 498 parents of children (aged 3–12 years) in France found an increase in children overeating and snacking more frequently during lockdown, as well as more home cooked meals and cooking more with children (9). Favorable changes were also reported in Greece, in a survey of 397 children and their parents; these included increased home cooking, reduced fast food, and increased fruit and vegetable consumption (10). However, increased body weight was found in 35% of respondents and this was associated with increased snacking, as well as decreased physical activity (10). Another study, from Italy and involving 41 children (aged 6–18 years) with obesity, found unfavorable lifestyle changes during lockdown, such as increased sugary drink intake and decreased physical activity (11).

Internationally, much of the research into the impact of COVID-19 on dietary habits has been through cross-sectional questionnaires. Few qualitative studies have explored families' experiences; one Californian study involving 48 parents of children (5 to 18 years) found changes in eating habits, increased snacking and more family connectedness at mealtimes (12). Another qualitative study with 25 primary food gatekeepers in Australia reported increased home cooking, enhanced food literacy and increased consumption of family meals (13). Further, there have been studies examining food intake in children with obesity (11), and surveys to examine children's eating habits (14). However research exploring children's experiences during lockdown, including children's food-related experiences, is lacking.

During the first national lockdown in the UK, children were at home with their family for a sustained period; the changes to their diet and food habits is not known. For example, the effects of changes to shopping, as well as spending more time within the home food environment, and with family members, is unclear. It is also important to note that children ordinarily spend a large proportion of the year in school, and school meals can make an important contribution to children's diets. Weight gain in children has been associated with school holiday periods (15, 16)–and indeed, a sharp increase in the prevalence of obesity is conspicuous in the National Child Measurement Programme in England for 2020/21, for 4–5 and 10–11 year olds (9.9% in 2019/20 to 14.4% in 2020/21 and 21.0% in 2019/20 to 25.5% in 2020/21, respectively) (17).

Food choice is multifaceted, complex, and dynamic. Given the multiple changes accompanying the national lockdown, changes to family food habits and food choice may be anticipated. Socioecological theory suggests that health-related behaviors such as food choice are influenced by factors both intrinsic and extrinsic to the individual (18). Levels of influence include: the individual, their attitudes, preferences and beliefs; interpersonal relationships with those closest to them such as families, friends and social networks; the community in which they live; and the wider society and public policy (19). Stressors related to COVID-19 such as prolonged lockdown, fear of infection, financial loss, lack of in-person contact with others, frustration and boredom have been reported (20–22); it is not known how these may have influenced families' food choice and dietary behavior during the COVID-19 pandemic. Likewise, the changes to food access due to government restrictions (for example less frequent food shopping and more food delivery) could influence family food choices during this time.

An important consideration in this is the home food environment, which is a series of interactive overlapping domains, relevant to dietary behavior (23). Influenced by social, cultural, political and economic factors, the domains have multiple contributions–including those most central to a child such as food availability, parental diet, practices and rules, and family eating patterns (24). Parents are crucial moderators of food within the home and, as nutritional gatekeepers (the person responsible for planning, sourcing and preparing family food), parents can contribute to creating environments that foster healthy eating behaviors or promote unhealthy choices and excess weight (25). Children have generally reported a lack of input when it comes to food purchasing decisions and that instead, their parents' financial and health concerns informed food shopping and ultimately choice (26). Other research however, has pointed to primary school-aged children as active decision-makers regarding food choice (27), and as agents of change influencing cooking and food choice at home (28) with growing authority over everyday food decisions (29).

We need to understand how COVID-19 and the restrictions of lockdown impacted families' food practices and food choice–in order to consider the implications and inform support for families post COVID-19. This study aimed to explore the food-related experiences and perspectives of families with primary school-aged children during the COVID-19 pandemic and the national lockdown.

## Materials and Methods

### Study Design

A qualitative methodology was chosen based on the study's emphasis on families' individual experiences, their household activities and how and why they behave in specific ways. This can enable a focus on participants' everyday life experience (30), and provide theoretically generalisable data to develop concepts and understand phenomena (31).

One-to-one interviews were chosen as they provide more detailed insight into participant experiences and a better understanding of their behavior, than focus groups (32). Semi-structured interviews were selected to allow for discussion and to pursue interesting and emerging themes in depth. Separate interviews for each parent and child of each dyad (pair of related individuals) was chosen to enable greater insight into family life during the pandemic, and to support participants talking more freely and with less influence. In particular, it has been suggested that a parent's presence in a child's interview may prevent them from being heard (33). Online interviews were planned, as it made sense during the time of social distancing, particularly as video communication to keep in touch with family and friends, for schooling, and for work had become more commonplace during the pandemic (34). Further, a researcher's remote presence and lack of obtrusive recording equipment can promote informality and a sense of ease, conducive to researcher-participant rapport (35).

An inductive approach for the data analysis was chosen to allow findings to emerge from the data's frequent or dominant themes (36). Reflexivity was embedded within the study design, to recognize the role of researchers in the process and to try to reduce the influence of preconceived opinions (31). Specifically, memoing and researcher discussions took place throughout the study design, data collection and analysis, and sources of bias were acknowledged and reflected upon. Ethical approval for this study was granted through the faculty research ethics committee.

### Recruitment and Participant Eligibility

Parent-child pairs were recruited through purposive sampling; parents were required to have spent the first lockdown at home, either working or not, with at least one child (Year 4 or 5; aged 8–10 years) who was not at school between 23 March and 8 September 2020. This age group was chosen as these children were not included in the phased reopening of schools in June 2020 (37) and therefore experienced longer periods of school closure. Parent-child dyads were recruited through a junior school in a market town in the north of England. The school had a larger than average cohort in target years 4 and 5 (8–10 years), and whilst the school was located within the 40% most deprived neighborhoods in England (38), <10% of pupils were eligible for free school meals, lower than the national average of 17.3% (39). Initial contact with the school was made by email and followed up with discussions with the school leadership team. Following school agreement to participate, all parents of year 4 and 5 pupils were invited to participate via a notification on the school's parent communication app. The notification provided outline details of the study and the researchers' contact details. Parents who expressed an interest were provided with further details and the information sheet for the study.

### Interview Schedules

Two interview schedules were developed: one for parents and one for children. The questions and associated prompts focused on families' experiences around food during COVID-19 and the first national lockdown period. Both the parent and child schedules explored food practices and dietary behaviors; questions related to topics, such as food choice, food shopping and preparation, food practices in the home, and food from outside the home. The questions were developed to be open, and non-leading, with particular attention paid to age-appropriate wording for the child interview schedule, e.g., Can you tell me what you remember about the first national lockdown when your school closed? What about food during lockdown, what do you remember about that? A final question gave participants the opportunity to introduce any information they thought was relevant but which had not come up. The interview schedules were reviewed by an expert panel of academics and public health practitioners who provided feedback on content, appropriateness of topics and the language use. Six pilot interviews were conducted with three parent-child pairs, and the interview schedule was refined to allow for more relevant discussion and to improve the question order. The interview schedules are available from the corresponding author upon request.

### Data Collection

Semi-structured interviews were conducted in November and December 2020 (during the second UK national lockdown). In total, 40 interviews were conducted, each with one parent or child from parent-child dyads, except for two interviews involving two siblings in the same household fitting the eligibility criteria. Interviews with parents and children lasted on average of 26 and 15 mins, respectively. Informed consent was gained and interviews were conducted remotely using video conferencing software (Microsoft Teams), with participants in their home. A researcher led the interviews using the interview schedule; however, this was flexible and depended upon the direction of discussions and topics arising. During each interview, the researcher noted non-verbal observations, salient points, and initial thoughts for inclusion in data analysis. Immediately following the interview, parents completed a short online questionnaire to collect demographic characteristics. Recruitment and interview of dyads was conducted until it was felt that data saturation had been reached, and there were no further issues or insights arising from the data.

### Data Analysis

Interviews were video recorded with the participants' consent, and then transcribed to a protocol using a denaturalised approach removing interview noise such as stutters and mannerisms (40). Transcripts were checked twice against the video recordings for accuracy, and then anonymised. The transcripts, researcher memos and observation notes provided the data for this study, and were imported into the software package, NVivo 12 Plus (NVivo 12 Plus, QSR International, Melbourne, Australia) to help with data management and data analysis. An inductive approach was adopted for the data analysis, which entailed data exploration, inductive coding and thematic analysis (36).

Coding was conducted to capture participants' attitudes, perceptions, and experiences related to food. This was an iterative process, and once all data had been initially coded, researchers reviewed and discussed the coding, before beginning another iteration. In all, four iterations of coding took place, with coding reviewed and refined with each successive iteration. Themes were identified from main concepts that appeared across multiple interviews and significantly contributed to understanding the research topic.

## Results

A total of 20 parents (19 mothers, one father) and 22 children participated in the study. Parents were aged 26–50 years, with the majority over 36 years. Children were aged 8–10 years, with most aged 8 years. All but one parent and child pair were white British, and all households were two-parent families, with most having two children. Participants lived in areas with an IMD decile 4–10 (10 being the least deprived), with 70% of participants living in areas with an IMD decile six or above, indicating lower deprivation levels (38), and four fifths of households had annual household incomes of £50,000 plus (80%). Demographic information for participants is provided in Table 1.

**Table 1:** Demographic characteristics of parent and child participants.

**Parents**			**Children**		
	***n***	**%**		***n***	**%**
**Sex**
**Female**	**19**	**95**	**Girls**	**11**	**50**
**Male**	**1**	**5**	**Boys**	**11**	**50**
**Age**
**26–30 years**	**2**	**10**	**8 years**	**14**	**64**
**31–35 years**	**1**	**5**	**9 years**	**4**	**18**
**36–40 years**	**5**	**25**	**10 years**	**4**	**18**
**41–45 years**	**9**	**45**			
**46–50 years**	**3**	**15**			
**Education**
**A-Level or equivalent**	**5**	**25**			
**Degree or equivalent**	**15**	**75**			
**Ethnicity**
**White-English/Welsh/Scottish/N Irish British**	**19**	**95**
**Mixed/Multiple ethnic background**	**1**	**5**
**Employment status prior to lockdown**
**Homemaker**	**2**	**10**
**Self-employed**	**1**	**5**
**Student**	**1**	**5**
**Working full-time**	**9**	**45**
**Working part-time**	**7**	**35**
**Employment status during lockdown**
**Furloughed**	**1**	**5**
**Homemaker**	**3**	**15**
**Student**	**1**	**5**
**Working from home**	**15**	**75**
**Household**		
	***n***	**%**
**Household income**	
**£15,000–£29,999**	**1**	**5**
**£30,000–£49,999**	**3**	**15**
**£50,000–£74,999**	**6**	**30**
**£75,000–£99,999**	**9**	**45**
**£100,000+**	**1**	**5**
**Household composition**
**2 adults, 1 child**	**3**	**15**
**2 adults, 2 children**	**11**	**55**
**2 adults, 3 children**	**4**	**20**
**2 adults, 4 children**	**1**	**5**
**3+ adults, 2 children**	**1**	**5**		

Data analysis provided four themes: commensality, elevated place of food in the home, snacking, and food shopping. Themes and associated sub-themes are presented in Table 2 and described below, with quotations to illustrate findings. Unique identifiers are assigned to participants, e.g., P1 and C1 are the parent and child participants, respectively, of one dyad (in the two instances where two children of the same parent were interviewed these are denoted, e.g., C6a, C6b and P6).

**Table 2:** COVID-19 and family food during the national lockdown: the four emergent themes and associated sub-themes, pointing to enhanced food literacy.

** 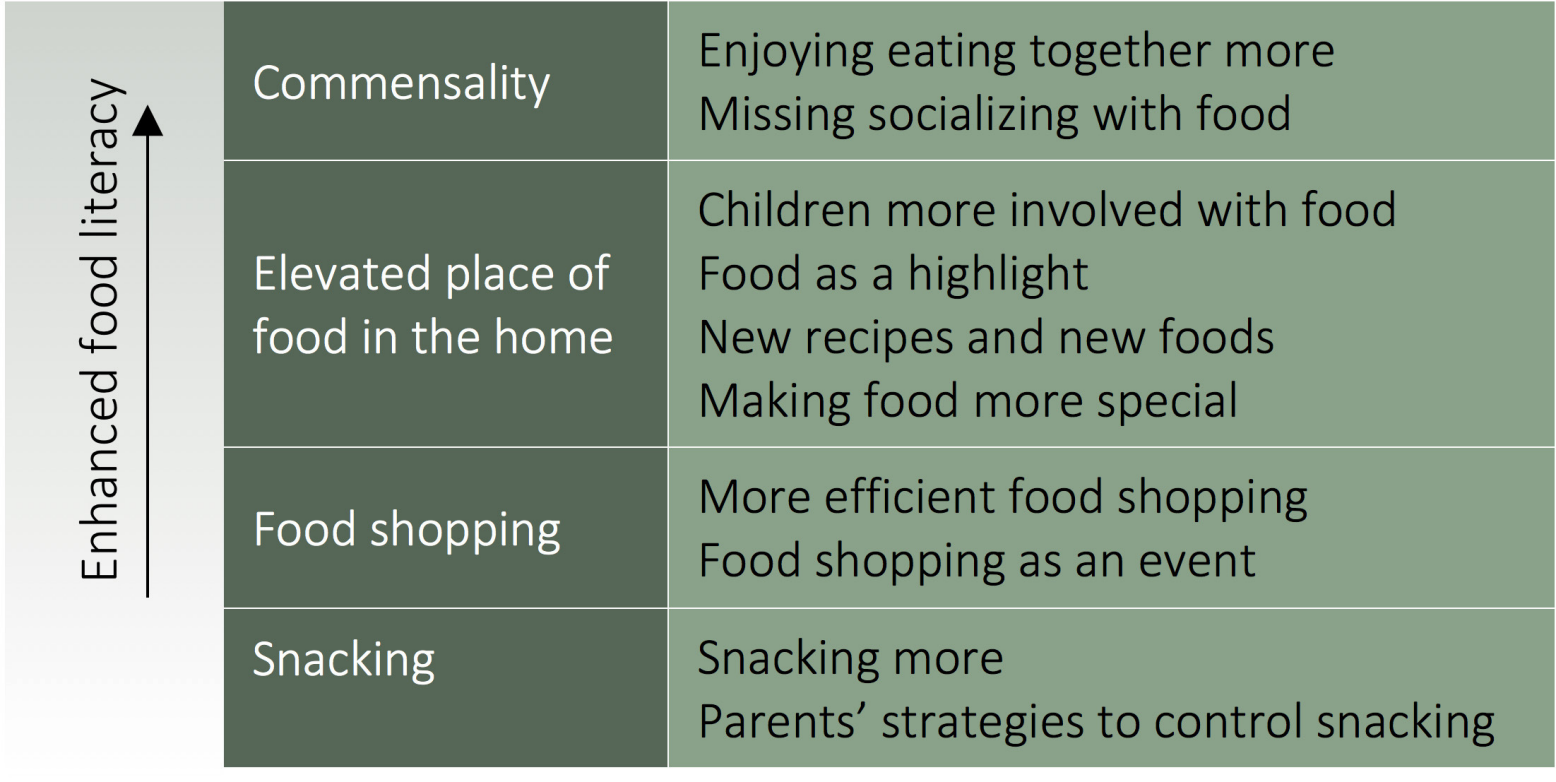 **

### Commensality

Many participants reported changes relating to commensality [a concept to describe eating with others (41)] during lockdown. Some recognized feeling positive about eating together as a family more, whilst others missed social interactions (which often involved food) with wider family and friends.

#### Enjoying Eating Together More

Children and parents alike recognized that they were spending more time eating together and enjoyed this. Sitting to eat together more regularly became important, and many reported mealtimes at home as a more social occasion.

*... in lockdown: breakfast, lunch and tea, it was all [of us eating] together–we made a point that no electronics [were out]... we're going to just sit and have this time... we might not ever get the time like this again, really (P13)*.

*I think we probably ate more together as a family because obviously we weren't... we're normally all here, there, and everywhere, and I think it probably meant that we actually spent more time eating as a family (P16)*.

*It [eating together] felt a bit more happy, happier than usual because we get to, we got to talk about, we got to talk about things and our worries (C1)*.

*It was nice to be with my dad... It was different because [usually] he works on weekends, and we're off. He doesn't work on a week, and we're at school. We don't really get to see him that much. I'm really glad that we get to see him more than we used to do before lockdown (C14)*.

Changes to mealtimes, often with parents eating their evening meal earlier than pre-lockdown, enabled families to eat together and was possible because after-school activities and work were no longer barriers. For children, large lunches (in school) and small convenient evening meals were replaced by smaller snack-type lunches and large, social evening meals. Interestingly, participants reported continuing post-lockdown to try to eat together as often as possible.

*Normally we would eat separate to the kids, just through work patterns and time I get home and stuff, but we were sitting down as a family and eating more together (P17)*.

*I think food has remained a social thing, in that it's what the four of us do, to have a focus point of the day and to sit down together (P11)*.

#### Missing Socializing With Food

As well as eating together as a family more, parents acknowledged missing eating with others, particularly extended family and friends.

*That [food] completely changed because on weekends [before lockdown], we were basically spending most of our time with friends. We were always eating out or eating at someone's house or someone was coming over to ours (P13)*.

*I missed the family, eating with the family. That was the biggest thing for me (P15)*.

In parallel, children enjoyed more family meals but missed their friends and missed eating with friends during the “noisy” social school lunch.

*It was quite weird: normally, I'm crowded with people around me... while I eat (C18)*.

*I missed having a really good chat. I always miss... seeing my friends, just seeing everyone eating and hearing the chatter of other people around me (C16)*.

*I was sad that I didn't get to see my friends because of lockdown. That made me sad, that I didn't get to see my friends. Also... I miss talking to them in the dinner hall (C14)*.

Interestingly, children also referred to returning to school post-lockdown, and having to eat lunch in the classroom rather than the dining hall and still not being “*allowed to move to where your friends are sitting”*.

### Elevated Place of Food in the Home

During the interviews, parents referred to spending more time on food-related activities, e.g., meal planning, shopping, preparing, cooking, as well as making food more “special” and using food as a source of enjoyment. Some parents recognized that they were becoming fatigued with the situation–and looked to “nicer food” and takeaways to try to alleviate the food monotony. Children also had more involvement in food preparation and expressed their opinions over food choice more often. Overall, it was apparent that food had acquired an elevated place within the home.

#### Children More Involved With Food

Children were more involved in cooking and baking; parents explained this as an activity to do while children were spending more time at home.

*I think a lot of the activities we did, looking back, revolved around food, even making it, or playing with it, or preparing it (P15)*.

*We did lots and lots of cooking. We cooked cakes and baked cakes... We'd make a lot of cakes, and we made… I remember we made loads of cheesecakes (C12)*.

*I think I did a bit more [cooking] because I didn't really have much time when it was not in lockdown, to bake because I only really had weekends off and so there's not really much time after school. So I think I did a bit more (C16)*.

Parents also spoke of children being more involved and wanting “to have a bit more of an input” in what they were eating, and playing a more central role in food decisions, with more freedom to choose. Children also reported this increased input into their food choice.

*I mean she [daughter] got a bit more picky about what she wanted. It was always* ***this*** *cereal bar, then she was like, “Oh, I want a fresh fruit salad” and, “I want this” and, “I want that”, and she was more specific about what she wanted (P3)*.

*It went from having some Weetabix before he [son] went to school to, “Well, I'll just have some boiled eggs on toast”, and “I'll just have some chopped up fruit while I'm waiting”, and “I'll just have this, and I'll just have that” (P4)*.

*He [son] had a lot more freedom with it [food] as well, because he'll just go and get something from the fridge (P8)*.

*Some days they'll [children] come up to me before I even start cooking and they were like, “Can we have this tonight?” or they knew if I was going shopping, they'd ask for a specific thing (P3)*.

A popular meal for children during lockdown was a “snacky lunch”, a variety of (typically) cold foods such as boiled egg, sliced meats, sliced/cream cheese and vegetables slices (e.g., peppers, carrots). For some children, snacky lunches were introduced during lockdown; for others already familiar with snacky lunches, these were eaten more often.

*At lunchtime, her [daughter] and Evelyn [other daughter] developed a meal called “Scraps and Pieces”, which sounds ridiculous, but basically, it was things like carrots, cucumber, houmous, celery, ham, all on a plate with different vegetables. That was their favorite thing and that's what they ate pretty much all of lockdown (P16)*.

*For lunch, we would probably have... a snacky lunch, where we would have, maybe a bit of ham, a bit of cheese, a bit of cucumber, a bit of tomato, and maybe a boiled egg (C19)*.

Interestingly, once lockdown ended and schools reopened, it emerged that more children took packed lunches than before. Children felt that packed lunches gave them more input into their food, or felt that it was easier than consuming school lunches in the classroom (which was the practice when they returned to school post-lockdown).

*I can choose what I put in [my packed lunch], instead of having the same thing (C2)*.

*She [daughter] wants to be independent... that's why she wants to take her lunch with her (P15)*.

*Since she's been back she's opted for packed lunches only now, because she hates having to go carry the tray back [to the classroom], because she's afraid she'll drop it (P10)*.

#### Food as a Highlight

It became evident that food became a primary source of enjoyment for many participants, who acknowledged it was one thing they could still influence and choose to enjoy.

*It [lockdown] was just so boring and so mundane that we needed something to look forward to. You look forward to food because... we enjoy it; our family, we enjoy food. It was something to be excited about… like, look forward to, during the day (P13)*.

*It was one of the main purposes of the day sometimes, which sounds ridiculous, doesn't it? But it was that one thing of... we'll get to sit down together tonight and eat a really nice meal (P11)*.

#### New Recipes and New Foods

Parents reported enjoying spending more time cooking and preparing family meals from scratch, explaining that they did not have time to do this pre-lockdown. Interestingly, parents felt that their children tried more new food, and that generally, there was more variety in what was eaten by the family, with different recipes and new foods.

*I think the main thing for me has been just having that time now to be able to prepare food, nice meals for them, rather than always thinking, “Oh, God, they're eating crap all week!” (P17)*.

*I was cooking more like my mum used to cook, or my grandma used to cook (P13)*.

*I think I put a lot more thought into trying new foods and different recipes that we hadn't done yet (P9)*.

Overall, parents and children felt that the family was eating healthier meals, with less eating out, fewer convenience foods as well as more cooking from scratch. Many parents acknowledged feeling good about this, satisfied to be providing “better” family food.

*We cooked more healthy things. In our curries, we normally have loads of meat–but during lockdown, we had lots of vegetables (C13b)*.

*We couldn't go out to restaurants, so we had to eat inside, and I think we ate healthier snacks (C19)*.

*I made sure that we had plenty of fruit in and that the meals were healthy and they were cooked from scratch rather than convenience food and things like that (P11)*.

*I have got that bit more time to meal plan, and make sure they're getting a probably more balanced range of meals... than they were before (P17)*.

Some participants also reported that some of these changes had persisted post-lockdown, and for example, felt like they were still eating healthier meals compared to pre-lockdown.

*We still do meal plan, and we do still try to have a healthy tea as such, with vegetables. We're still making things pretty much from scratch, when it comes to certain meals (P7)*.

*In lockdown we did try and make foods that we really enjoy. We've been trying to get those recipes back and trying them again (C13b)*.

#### Making Food More Special

Food became a focal point in the home, and parents reported spending more money on food and buying nicer food and treats. This was explained by not being able to eat out, having nothing else to do or to spend money on during lockdown.

*The one enjoyment was: let's plan a really nice meal, that's healthy, that's really tasty but guilt-free and we can all sit down and enjoy that meal (P11)*.

*Just trying to think of different recipes to cook and things that were, maybe a little bit more special and to make you feel like you had... a change of the routine (P20)*.

Parents acknowledged that, over time, food in the home became monotonous; and they were “*fed up of thinking and having to organize something every mealtime”*–opting for more takeaways, premium foods and eating outside to make mealtimes more interesting. Children recognized these changes too.

*I think the longer that [lockdown] went on, it became a bit... frustrating, because we were just constantly having to cook and that probably also impacted on our creativity because we just got bored with it (P19)*.

*We stopped really eating out because everywhere was shut, but we had takeaways and brought them home, so that was good (C16)*.

*I mean, we had a lot of fast food in lockdown, we didn't have a lot before (C4)*.

*The girls quite liked having a picnic outside on the grass; even though it's just in the back garden, it felt like it was a bit special (P16)*.

*We let the kids eat outside a lot more. I think that was just a combination of trying to get them outside and a bit of fresh air–also, just a different location to eat (P19)*.

*In the summer we ate quite a lot of meals outside on an evening. We spent a lot of time in the garden (C11)*.

According to parents, premium foods and takeaways also endured post-lockdown, displacing eating out to some extent.

*We still don't eat out, still can't get my head round eating out very often. I don't like it, although we've done it a couple of times. We do now get takeaway. We probably now have takeaway once or twice a week (P18)*.

*It's [takeaway] like us treat for the week, and it's something to look forward to. Frankie [son] absolutely loves it, so I've just carried on, and it's easier as well; I don't have to start cooking then [...] We're still getting us takeaways really. It's as if it's like your bit of enjoyment of the week as well (P8)*.

### Snacking

Parents felt that they and their children snacked more. Increased snacking was also recognized by children in this study, and parents looked to implement strategies to control the number of snacks eaten by children.

#### Snacking More

Parents felt that children ate more snacks and talked about their children being hungry and asking for more food. The emphasis was on the requests for more food, rather than specific types of food, although common foods mentioned by parents included crisps, biscuits and fruit. Parents also commented that this was not “real” hunger, but instead, driven by boredom and availability of food in the home.

*Yes, they had a lot of snacks [in lockdown]... I think they were hungry all the time. I don't think they actually were hungry; I think they were just bored (P16)*.

*The thing with him [son] was that he was constantly saying that he was hungry. I think that's by being at home and knowing that there's a cupboard there that's full of food, that he can just nip in and get something (P6)*.

Children also recognized that they were “*snacking a bit more”* during lockdown.

*Maybe, [snacked more] just because we didn't have to sit and just do our work, because if we were at school… and we had more freedom to go to the toilet more often and... to waste school time [laughs]. Olivia [sister] definitely snacked a lot more (C11)*.

*Well, I would have more time to have them [snacks] because I wouldn't be at school or at gymnastics. Also, [pre-lockdown] I would be at school a lot, so I wouldn't have time to eat lots of things... except dinner at school (C14)*.

During the discussions, it was evident that most “snacks” were foods that were high in fat, sugar and salt, although some parents and children also reporting eating more fruit as a snack.

*I've never bought so much stuff for them [children] to snack on. I've had a chocolate sweet tub which I've never had before (P7)*.

*We ate a lot more fruits. Sometimes, we'd have a little snack in the sweet cupboard; apart from that, it was mainly fruits (C13)*.

Boredom was reported as a driver for increased snacking amongst parents, alongside wanting to treat themselves. For some, this was explained as a coping strategy for the ongoing stress and worry caused by the pandemic. For some parents, alcohol consumption increased for the same reasons.

*Probably a little bit panicked about everything, so I'd probably just ate out of... emotion. I don't know, it were just a strange time (P14)*.

*I think that [stress and anxiety] had a part to play, and I think as well, it was boredom. Knowing that we were having some treats that night or sharing a bottle of wine and some dips and crisps was like the highlight of the day (P15)*.

#### Parents' Strategies to Control Snacking

Parents acknowledged implementing strategies in response to their children's increased snacking. These included straightforward steps such as hiding the snacks away and offering healthier alternatives, as well as other ideas they had found online, such as putting out snack boxes or devising “price lists” to limit the amount eaten. Snacks were also used in some instances by parents, to incentivise children to complete tasks during the day.

*I became a lot more aware of the amount of snacks the kids were having. I looked for tips, from other parents as to how they were doing it [limiting snacks] (P15)*.

*I made a shop menu board, so they had to–each snack had a value, a price value–they had a pound a day to spend on snacks. Once that pound went, then they had no more snacks (P7)*.

*Once Mummy made a massive price list and we had to pay for our sweets (C7)*.

*Sometimes I did use it [snacks] as an incentive as well. If he [son] asked for some food, I would say, “Well, just do this piece of writing, and then you can have a snack” (P20)*.

Parents acknowledged that concern over their child's reduced physical activity and potential weight gain during lockdown, partly drove their desire to try to control their increased food intake.

*So I'm never concerned about what she [daughter] eats because she does lots of sports, in and out of school. But during lockdown again she seems to have quite healthy appetite and I did sort of think, “Oh, how will this work, with her not exercising as much?” (P1)*.

### Food Shopping

As well as altered food habits at home, shopping habits and routines also changed, with families introducing new and more efficient shopping. Participants' attitudes toward food shopping adjusted, in part, because of the changes to the retail food environment.

#### More Efficient Food Shopping

Parents spoke about the importance of planning meals and avoiding having to shop too often, and no longer 'nipping' to the shops. Families stopped shopping together for food and it became the exclusive responsibility of one member of the household, with children no longer accompanying them. Many of these changes were due to the restrictions in place, as well as fear and minimizing risk.

*Typically, we'd sometimes go to the shop to pick up different bits and pieces during the week, but we stopped doing that (P19)*.

*I was really conscious: we really must try and get everything in this one shop, to reduce, really, the risk of going out and being with anybody else. I really try to get everything on that Tuesday morning shop (P5)*.

*Mum usually got really big shops, so it lasts quite a few weeks. It makes sure that we also have enough to last a couple of weeks, so we don't have to do a big shop the next day (C14)*.

*My mum might have been scared that if I went inside, I might catch Corona because of all the people (C19)*.

Participants also adapted their shopping; many tried food deliveries for the first time and those who struggled to get delivery slots at supermarkets looked to local shops that had introduced deliveries during lockdown. Participants also shopped locally because they wanted to support their local economy, it was easier, and they had more time.

*I think there was a couple of key things that changed after lockdown. I think one of the things was that we started ordering most of our food online. We stopped going to the shops just because of the risk factor (P19)*.

*It did [shopping changed] a lot because we started getting just a click and collect once every week, and normally, before it was like, we'd go into the shop and... go around more and... things like that (C11)*.

*One thing, actually, that we did do because we've had the time to do it, was to go to the local veg shops. I was buying more fresh fruit and veg (P6)*.

…*the queues to get in [the supermarkets] were huge, and the whole rigmarole, the one-way systems, queuing at the checkouts, and things like that; it was just easier to go local (P17)*.

Interestingly, participants reported a reduction in food waste, explained primarily through increased food planning, and because using up all of the food within the home became more important as participants tried to avoid shopping.

*I'd started having to get a bit creative of what we had left, like, “Oh, what can I put with this old spinach and this half of butternut squash?” or whatever (P5)*.

*By Friday, there's not a lot in except what we're going to be making maybe on the Saturday, and then we go shopping again. I think that our food waste has gone down quite a bit (P13)*.

Post lockdown, many participants reported resuming their usual shopping practices, e.g., nipping to the shops more, no longer shopping online–however, some continued shopping more locally as they felt the food quality was better, it was more reliable, or they wanted to continue to support local businesses.

#### Food Shopping as an Event

During the interviews, it emerged that shopping was no longer enjoyable for some participants due to the restrictions and also reactions from fellow shoppers. Participants acknowledged feeling guilty and embarrassed when they thought they were being judged for purchasing too much food.

*It [shopping] were just hard work, harder work than [pre-COVID]... It was an event in your life, rather than you just going and getting what you needed (P14)*.

*Everybody looked really angry, and quite aggressive. I couldn't get out of there fast enough. It was really depressing (P15)*.

*I was embarrassed as well... I kept saying, ”I'm not panic buying. It's just there's five of us. We're home all the time” (P5)*.

However, for some participants, shopping was a means of escape, somewhere they could leave the house for.

*Back then, it was a highlight because it got you out of the house, a bit of peace and quiet as well (P10)*.

*It was one of the few things, again, that you could do, wasn't it? I wasn't one of these people who avoided going to shops or supermarkets, for fear of catching COVID (P17)*.

## Discussion

This study revealed the food-related experiences and behaviors of families with primary school-aged children, during COVID-19 and the first national lockdown. As the government's policies restricted usual activities, families spent more time together over food, enjoying family meals, cooking more meals from scratch and involving children in cooking. The place of food within the home was elevated as it became a source of enjoyment, entertainment, and comfort in uncertain times. Food choice was less focused on convenience, and participants spent more time on food, e.g., planning, shopping, preparing, cooking, as well as buying more premium foods and making food more special.

It was evident that there were changes in commensality; parents and children ate together more often, and there was a shift in food routines, with more quality family time during meals. Eating together as a family also adjusted the food and meals eaten by participants with, for example a more substantial and leisurely evening meal. Increased commensality corresponds with a Canadian study (conducted in April-May 2020) of families with 4–8-year-olds, where parents ate together more with their children since COVID-19 (8). Likewise, a US study reporting similar findings, attributed this to increased parental presence in the home (42). Other studies (from the US and Australia) have reported increased family mealtimes and family connectedness over food during lockdown (12, 13). Such changes (including those observed in the present study) are important, not least because they afford the opportunity for parents to model good dietary habits, relevant in promoting for example, children's acceptance and willingness to try new foods (25); the potential for less favorable eating habits also applies however.

As well as favorable changes such as spending more time cooking and preparing meals from scratch, unfavorable changes also emerged. These included increased snacking. This has been reported previously in adults (43–47) and children (8, 10, 12, 43), including in UK surveys (48, 49). In the present study, increased snacking in children was attributed to boredom and being in an environment with ready access to food. This is in line with previous research which found that snack frequency in children was predicted by boredom, and children attempted to fill time or seek comfort from food during lockdown (9). Children's increased snacking warrants further attention, given the potential effects on nutritional status. Ignoring internal cues of hunger and instead, eating through boredom over a long period can induce weight gain (50). This is particularly pertinent given that predictions are that childhood obesity levels will increase due to the pandemic and may not be easily reversible (11, 51), and substantial increases in childhood obesity were reported for the 2020/21 school year in England (17).

Some parents attributed their own increased snacking to pandemic-related anxiety or worry. A study from New Zealand (47) highlighted adults' increased consumption of snacks during lockdown, and an overall shift to an unhealthy dietary pattern, with authors pointing to the need to mitigate stress in future responses by government and employers. More generally, snacking has been related to opportunity-induced eating and coping with negative emotions (52), with emotional eating theory, suggesting negative emotions induce eating, as eating can reduce negative feelings through psychological and physiological mechanisms (53). Related to COVID-19 and the restrictive measures, increased stress and a decline in mental health has been recognized – with a deterioration in mental health in UK adults as the pandemic emerged in spring 2020 (20). Previous work has indicated that COVID-19 restrictions and fears over illness potentially increased anxiety and lead to stress-related eating, particularly foods high in sugar and fat (54). A UK survey found that during the pandemic, adults ate more chocolate, cakes or biscuits, and crisps when feeling tired, stressed, bored or anxious (55).

Interestingly, many parents in the present study attempted different strategies to control children's snacking, including restriction, providing healthier alternatives, and other means such as a snack menu board with limited “money” to spend. Parents also reported using food as an incentive or to control behavior. Previous research from the US reported various parenting practices during the pandemic, such as more snack planning and more emotion-based snack feeding, and exerting more control when experiencing high-stress levels (42). Further, increased stress levels in parents during lockdown in a French study were found to predict greater increases in giving children autonomy regarding how much to eat and, according to the researchers, parents may have become too permissive regarding the food offered (9). Supporting parents to promote good dietary practices and routines is important, with for example, an understanding that children learn to positively associate food with feelings generated from a reward, and that this might affect overeating and weight gain (56, 57). Likewise, support regarding food parenting practices is important alongside an appreciation that the extremes of complete coercive control or full relinquishment of control to the child can have negative effects, with the ideal being control through guided choice (58).

In this study, parents recognized children's increased food choice autonomy; children chose their own and their family's food more often and were more involved in food preparation. The parents in this study also reported involving their children in meal preparation more, which children enjoyed. It is interesting to note that some of the changes arising out of lockdown, for example, child involvement in meal preparation, which has been associated with healthier diet consumption (59), have the potential to promote healthy eating. Interestingly, an uptick in packed lunches (when schools reopened) was apparent in the present study; this may be related to changes with how school lunches were provided at this time, as well as greater food choice autonomy during lockdown, and children wanting to maintain more input into their lunch.

Food became more important to families in this study, and parents spent more time planning and preparing meals (including from scratch) and sought new recipes; they also gained satisfaction in spending time providing “healthier” meals to the family. Increased planning and preparation of home-cooked meals and cooking from scratch (8, 9, 13, 60–62) and perceived healthier eating during lockdown have been found in other studies (8, 63). Likewise, a UK study examining food choice motivations during the pandemic found that ease of preparation became less important, and family involvement more important to parents and carers (64).

Over time however, food preparation became monotonous, and for a break from the usual routine and to make mealtimes more enjoyable, participants bought more premium foods and had takeaways more often. Increased takeaways during lockdown due to boredom with your own home-prepared food has been reported elsewhere (13). This differs from a survey which reported a decrease in takeaway food consumption during lockdown (65); however, only 27% of survey respondents had children under 16 years, and it is possible that having to prepare family meals for children, as in the present study, led to more fatigue in this respect, driving takeaway orders.

As the pandemic continued and food businesses reopened, participants did not resume their pre-lockdown rates of eating out, and instead focused on takeaways. Parents felt it was not as safe or as pleasant to eat out due to the restrictions in place. This corresponds to findings from a US survey (administered in October 2020) of families (61) reporting that takeaway was perceived as safer (than eating in restaurants), and increased during COVID-19. Takeaway food has been found to be excessive in portion size, energy, macronutrients, and salt (66). The impact on nutritional status of increased takeaways during the pandemic and beyond should be examined, including consideration of their replacement of eating out.

In the present study, parents reported using online shopping and deliveries because of changes in the retail food environment and government restrictions. In the UK, reduced food availability in shops influenced food choice during the pandemic, and food systems were initially put under pressure as consumer purchasing habits changed (67). Periods of panic buying resulted in stockpiling and hoarding, which resulted in shortages of some items (68). Online food shopping increased, as many tried to avoid exposure to other people (69, 70). A relocalisation of food was also evident in this study, with participants turning to local food retailers and shopping more locally, reflecting trends for more localized food purchasing during the lockdown, as reported by the Food Standards Agency (49). Likewise, UK consumer data has revealed sales of food for home consumption increased, out of home food sales reduced, with increased use of local food retail (71, 72). Localized food shopping has also been reported in Italy (73) and in France (9). Further, it has been suggested that relocalisation was likely to be sustained with the work from home and distancing restrictions in place (67).

As well as shopping more locally, participants shopped as infrequently as possible, discarding the usual “nipping to the shops”. This was driven by fear of visiting shops too often, and similar findings were reported in an Australian study (13). Avoidance of shopping also led to reduced household food waste, as participants delayed needing to shop by saving and reusing leftover food. Reduced food waste was also attributed by participants to meal planning and list-making. Previous research from Spain has reported that reduced food waste during the COVID-19 lockdown was related to reduced shopping frequency, improved food management and preparing more creative recipes with leftovers (62). Likewise, changes in household food management and reduced food waste has been reported in Italy (73, 74) and New Zealand (75). Further, participants in the present study discussed food waste in relation to concerns over food access and availability, corresponding with a Tunisian study (76) which suggested that reduction in food waste during COVID-19 lockdown was likely to be driven by socioeconomic factors rather than environmental concern.

Interestingly, many of this study's insights suggest that the stay-at-home policies in place during the national lockdown may have provided the opportunity for improved food literacy. Many changes reported by participants (such as cooking from scratch more, seeking different recipes and trying new foods and better food management) point to spending more time with food, becoming more skilled in food preparation, and gaining more knowledge about food. Likewise, a greater ownership of food and feeling good about providing healthier meals for the family, as well as more localized food shopping may propagate a deeper understanding of the importance of food and its role in social relationships with others and thinking about where food comes from, when considering supporting the local economy. Many of these relate to the components of food literacy, such as the planning and management, selection, preparation, and eating of food (77), and food-related skills and knowledge (78). Food literacy is important for individuals to navigate the complex food systems and can help to ensure food intake is in line with nutritional recommendations. The proposed change in food literacy in this study is supported by evidence from other research. In a study of 38 countries (79) food literacy behaviors of planning, selecting and preparing healthier food were higher during COVID-19 restrictions. Likewise, a cross-sectional study (80) reported that Canadians became more food literate (trying new recipes and new ingredients) since the pandemic. Many of the changes reported in the present study and the increased emphasis on food can be viewed overall as positive, however given the complex relationship between food and eating behavioral disturbances, this may not be the case for all individuals.

COVID-19 restrictions presented challenges to participants' usual food-related practices. Closure of food service outlets (e.g., cafes, restaurants) emphasized food preparation and consumption at home. Shortages of certain foods and changes to the retail food environment contributed to the adoption of new shopping behaviors. Participants recognized increased snacking for themselves and their children, attributing the latter to boredom or food availability at home. Government restrictions removed social interaction over food with, for example friends, peers, colleagues and extended family. The various influences on participants' behavior correspond to different levels of the socioecological model (18), for example more proximal influences affecting snacking, whilst distal influences at a policy level with respect to food shopping restrictions and reduced commensal eating with friends and colleagues. The pandemic's influence on diet and nutrition has been reported to have gone beyond the individual and community, reaching national and global levels of the ecological model (81). Further, it is proposed that behavior will have been unusually influenced by more distal levels than would be expected in other food choice transitions, given the nature of the pandemic and national lockdowns.

### Strengths and Limitations

The study's strength comes from incorporating both parent and child perspectives and experiences during lockdown, providing a rich picture of the home food environment at this unique time. Using remote video interviews widened participation to individuals who may have otherwise had time or place constraints. Conversely, it may have inadvertently excluded participants with limited access to the technology or competency required to participate (82). Further, whilst verbal and non-verbal cues are detectable; it can be challenging for the interviewer and the interviewee (83) particularly considering subtle non-verbal cues, and the reduced frame of a headshot restricting observation of participants' body language (84).

The limitations of this study should be acknowledged, and these include sample bias, e.g., potentially higher response rate from families interested in food or with healthier eating habits. The study data is derived from participants' recollections of lockdown and could be subject to recall bias. This bias may be more pronounced with child participants whose recollection of lockdown was not as clear as parents. Further, researcher-led interviews may have led to social desirability bias with participants saying what they think the interviewer wanted to hear.

In considering this study's findings attention should be given to the demographic characteristics of the sample population, which was almost exclusively mothers (95%), white British (95%), and educated to degree level (75%). Although there was a range of household incomes, half of households were earning £75,000 plus, i.e., at least twice the average household income in the UK. This is particularly relevant, given for example, better education levels and higher household income have been shown to be relevant to better food literacy skills (85, 86). Further studies to explore perspectives and perceptions of families from different backgrounds and with different demographic characteristics (such as ethnicity, education, household income) would be valuable, particularly as responses to COVID-19 may be sensitive to socioeconomic characteristics.

The potential impacts of changes on nutritional status, for example cooking from scratch, snacking, should be explored. Greater understanding of both favorable and unfavorable shifts to dietary behaviors will be valuable in informing public health interventions targeting families. Special consideration should be given to how lockdown may have impacted food literacy, and if learnings can be applied beyond the pandemic to improve food literacy and nutrition status for families. Incorporating, for example, food skills, cooking confidence and nutrition education, within efforts to support individuals to implement or develop some of the positive aspects seen in this study, is worthy of further examination.

It is unclear whether the effect of further lockdowns and the continuing pandemic will result in longer term shifts in behavior, and for example more reliance on the local food retail sector may persist. It may be that some of the changes (e.g., shopping frequency) initially driven by fear, apparently subside with usual habits returning. Further research, including across socioeconomic groups, is necessary to ascertain the longevity of changes and to understand any lasting shifts in behavior. This is particularly relevant given the nutritional implication of some changes (e.g., increased snacking) and the possibility of working from home and flexible working in the future.

## Conclusions

To conclude, this study has revealed how, as the government's lockdown policies restricted usual activities, families spent more time together over food, enjoyed eating family meals together, cooked more meals from scratch and involved children in cooking more. As well as favorable changes reported by parents and children (spending more time together, more time cooking and preparing meals from scratch, more efficient shopping, and less food waste), unfavorable changes also emerged including increased snacking. The place of food was elevated in families' homes and overall, findings point to improvements in food literacy. The longevity of changes should be investigated, particularly given the potential implication on the nutritional status of families.

## Data Availability Statement

The datasets presented in this article are not readily available because of ethical restrictions.

## Ethics Statement

Ethical approval for this study was granted through the Faculty Research Ethics Committee. Parents provided their written informed consent to participate in this study, and for their children to participate in this study.

## Author Contributions

HE conceived the study. LS wrote the original manuscript. HE and LS designed the study, conducted the data collection, analysis, reviewed, and approved the final manuscript. Both authors contributed to the article and approved the submitted version.

## Conflict of Interest

The authors declare that the research was conducted in the absence of any commercial or financial relationships that could be construed as a potential conflict of interest.

## Publisher's Note

All claims expressed in this article are solely those of the authors and do not necessarily represent those of their affiliated organizations, or those of the publisher, the editors and the reviewers. Any product that may be evaluated in this article, or claim that may be made by its manufacturer, is not guaranteed or endorsed by the publisher.
